# Low Soluble Syndecan-1 Precedes Preeclampsia

**DOI:** 10.1371/journal.pone.0157608

**Published:** 2016-06-14

**Authors:** Robin E. Gandley, Andrew Althouse, Arundhathi Jeyabalan, Julia M. Bregand-White, Stacy McGonigal, Ashley C. Myerski, Marcia Gallaher, Robert W. Powers, Carl A. Hubel

**Affiliations:** 1 Magee-Womens Research Institute, University of Pittsburgh, Pittsburgh, Pennsylvania, United States of America; 2 Department of Obstetrics, Gynecology & Reproductive Sciences, Division of Maternal Fetal Medicine, University of Pittsburgh, Pittsburgh, Pennsylvania, United States of America; 3 Clinical and Translational Research Institute, University of Pittsburgh, Pittsburgh, Pennsylvania, United States of America; Medical Faculty, Otto-von-Guericke University Magdeburg, Medical Faculty, GERMANY

## Abstract

**Introduction:**

Syndecan-1 (Sdc1; CD138) is a major transmembrane heparan sulfate proteoglycan expressed on the extracellular, luminal surface of epithelial cells and syncytiotrophoblast, thus comprising a major component of the glycocalyx of these cells. The “soluble” (shed) form of Sdc1 has paracrine and autocrine functions and is normally produced in a regulated fashion. We compared plasma soluble Sdc1 concentrations, in relation to placental Sdc1 expression, in uncomplicated (control) and preeclamptic pregnancies.

**Methods:**

We evaluated soluble Sdc1 across uncomplicated pregnancy, and between preeclamptic, gestational hypertensive and control patients at mid-pregnancy (20 weeks) and 3^rd^ trimester by ELISA. Placental expression level of Sdc1 was compared between groups in relation to pre-delivery plasma soluble Sdc1. Participants were recruited from Magee-Womens Hospital.

**Results:**

In uncomplicated pregnancy, plasma soluble Sdc1 rose significantly in the 1^st^ trimester, and reached an approximate 50-fold increase at term compared to post pregnancy levels. Soluble Sdc1 was lower at mid-pregnancy in women who later developed preeclampsia (P<0.05), but not gestational hypertension, compared to controls, and remained lower at late pregnancy in preeclampsia (P<0.01) compared to controls. Sdc1 was prominently expressed on syncytiotrophoblast of microvilli. Syncytiotrophoblast Sdc1 immunostaining intensities, and mRNA content in villous homogenates, were lower in preeclampsia vs. controls (P<0.05). Soluble Sdc1 and Sdc1 immunostaining scores were inversely associated with systolic blood pressures, and positively correlated with infant birth weight percentile.

**Conclusion:**

Soluble Sdc1 is significantly lower before the clinical onset of preeclampsia, with reduced expression of Sdc1 in the delivered placenta, suggesting a role for glycocalyx disturbance in preeclampsia pathophysiology.

## Introduction

The syncytiotrophoblast is a specialized, multinucleate epithelium formed by the fusion of cytotrophoblasts within the placental villi. With its continuous exchange surface interfacing with maternal blood, the syncytiotrophoblast microvillous membrane has critical functions including transport of nutrients to the fetus [1]. Deficient uterine spiral artery remodeling (failure of spiral arteries to transform to high-flow, low-resistance conduits), frequently seen with preeclampsia or fetal growth restriction (FGR), is thought to result in reduced or intermittent blood flow to the intervillous space leading to syncytiotrophoblast dysfunction and damage [2].

The surface of eukaryotic cells has a hydrated matrix of specialized proteoglycans and glycoproteins, linked to the plasma membrane, known as the glycocalyx [3,4] The vascular endothelial glycocalyx is a critical regulator of vascular function, and endothelial glycocalyx damage has been implicated in reperfusion oxidative injury, inflammation, atherosclerosis and diabetes [3,5–10]. The syncytiotrophoblast microvillous membrane surface also has a substantial glycocalyx [11–13] but its functions are poorly understood.

Syndecan-1 (Sdc1; CD138) is a major transmembrane heparan sulfate proteoglycan expressed on the surface (glycocalyx) of epithelial cells, endothelial cells and plasma cells[14,15]. Sdc1 regulates diverse cell behaviors including adhesion, proliferation, motility, intracellular signaling, growth factor and macromolecular cell surface binding and cellular internalization, angiogenesis, lipid metabolism, wound healing, regulation of leukocyte migration, and endothelial responses to fluid shear stress [14,16–20]. Sdc1 is strongly expressed on villus STB microvillous membrane, but not on cytotrophoblast or stroma, extravillous trophoblast, nor fetal vascular endothelium [21–27]. Expression of Sdc1 on the STB microvillous membrane is reportedly reduced in preeclampsia [21,22,25] and iatrogenic IUGR with fetal compromise[26], compared to uncomplicated pregnancy.

Extracellular domains of Sdc1 are constitutively shed from the glycocalyx, typically in processes regulated by sheddases (e.g., matrix metalloproteinases and heparanase)[3,28]. The soluble form of Sdc1 is thought to have important functions as a paracrine and autocrine effector, and competitor of transmembrane Sdc1 for growth factors and other extracellular ligands [14,15,20]. Sdc1 shedding is an important response in wound healing with effects on inflammation, proliferation and remodeling [3,15,20]. Circulating soluble Sdc1 concentrations can be increased by excess reactive oxygen species or inflammatory stimuli, as exemplified by the extremely high values observed with sepsis [3,10,28,29].

Soluble Sdc1 concentrations have not been systematically examined in uncomplicated (normal) or preeclamptic pregnancies. Our initial hypothesis was that plasma soluble Sdc1 levels would increase with advancing normal pregnancy, and increase significantly more so before and after the onset of preeclampsia in association with reduction of Sdc1 immunoreactivity on STB of villous placenta. The rationale was based on the concept that the increased systemic inflammation thought to underlie the pathogenesis of preeclampsia, representing an accentuation of the normal inflammatory response to pregnancy [30], might be a stimulus for increased Sdc1 shedding. We first measured soluble Sdc1 longitudinally in maternal plasma from uncomplicated pregnancies, by trimester and proximally postpartum. We then investigated if concentrations of soluble Sdc1 were different at 20 weeks’ gestation, before clinical onset of disease, and late pregnancy after disease onset, in women who later developed preeclampsia compared to normotensive controls and women who later developed gestational hypertension (without proteinuria). In a different set of preeclampsia cases and controls we compared villous placental Sdc1 expression profile, and looked for correlations with clinical variables and plasma Sdc1. Finally, to test for lasting constitutional differences, we measured plasma soluble Sdc1 one year after delivery, comparing non-pregnant women with a history of preeclampsia to women with a history of uncomplicated pregnancy.

## Methods

### Comparison groups

We conducted a nested, case-control study of pregnant women enrolled between 1999 and 2012 as part of an ongoing longitudinal and cross-sectional investigation of preeclampsia (Pregnancy Exposures and Preeclampsia Prevention Study; PEPP) at Magee-Womens Hospital and Magee-Womens Research Institute, Pittsburgh, PA, USA. Written informed consent was obtained from all study subjects. The University of Pittsburgh Institutional Review Board approved the study. The study participants had also given permission to be contacted for additional preeclampsia-related research studies upon consenting to participate in the PEPP Project. A subset women enrolled in PEPP were thus evaluated 1 year after pregnancy, with separate University of Pittsburgh IRB approval and written informed consent.

We studied 5 different subsets of patients as follows. These groups were mostly non-overlapping, reflecting comparison criteria.

1. We evaluated gestational changes in soluble Sdc1 using longitudinal maternal plasma samples from 8 women with uncomplicated pregnancy, at gestational weeks 7–11, 17–26, 37–41; and 24–48 hours postpartum and 4–9 weeks postpartum. Patient characteristics are given in S1 Table.
2. Comparisons of plasma soluble Sdc1 at mid-pregnancy were performed. Nine women in this study set developed preeclampsia later in the pregnancy, 9 developed gestational hypertension (without proteinuria) later in the pregnancy, and 19 had an uncomplicated pregnancy outcome (characteristics summarized in Table 1). The samples were matched for gestational age (mean gestational weeks ~20, range 18–24). Gestational age-matched 3^rd^ trimester samples were not sufficiently available from these patients.
3. We compared 17 women with preeclampsia to 17 with uncomplicated pregnancy, and 8 women with gestational hypertension to 8 women with uncomplicated pregnancy, during the 3^rd^ trimester after disease onset (described in S2 and S3 Tables). Fifteen of 17 women with preeclampsia delivered pre-term whereas all of the women with gestational hypertension delivered at term. Therefore, women with preeclampsia were matched by gestational age to controls who delivered at term but had provided earlier 3^rd^ trimester samples at the time of routine prenatal care visits. Women with gestational hypertension were matched by gestational age to a different set of controls with samples obtained immediately before delivery.
4. Both villous placental tissue and maternal plasma samples were obtained at the time of delivery from 19 women with preeclampsia and 25 women with uncomplicated pregnancies (samples not matched for gestational age at delivery). Clinical characteristics are given in Table 2. Two preeclampsia cases and 2 controls were also in study set #3, above.
5. We evaluated soluble Sdc1 levels in plasma from a separate set of 17 women with a history of preeclampsia and 19 with history of uncomplicated pregnancy, 1 year after delivery (S4 Table).

**Table 1:** Clinical characteristics of uncomplicated pregnancy control (n = 19), gestational hypertensive (n = 9), and preeclampsia (n = 9) groups; for comparison of soluble Sdc1 in gestational age-matched, 2^nd^ trimester maternal plasma.

	Uncomplicated Pregnancy (n = 19)	Gestational Hypertension (n = 9)	Preeclampsia (n = 9)
Age (years)	24 (20–35)	22 (19–36)	24 (17–41)
BMI pre-pregnancy (kg/m^2^)	29 (23–36)	26 (22–37)	31 (23–45)
Gestational weeks at venipuncture	19.7 (17.7–23.9)	19.9 (17.7–24.4)	19.4 (18.4–22.3)
Gestational weeks at delivery	40 (29–42)	40 (38–41)	38 (30–40) ^a^^,^^c^
Early gestational BP (<20wks.):			
Systolic (mm Hg)	114 (99–126)	119 (111–137)	116 (109–130)
Diastolic (mm Hg)	69 (59–80)	73 (64–86)	70 (68–78)
Pre-delivery BP:			
Systolic (mm Hg)	126 (104–138)	144 (141–163) ^b^	148 (141–193) ^a^
Diastolic (mm Hg)	73 (55–80)	90 (78–94) ^b^	97 (89–114) ^a^
Birth weight percentile	75 (30–91)	41 (16–60) ^b^	23 (1–93) ^a^
Uric acid (mg/dL)	—	4.8 (3.7–5.4)	5.9 (5.1–8.7) ^c^
Cigarette smokers (n, %)	0 (0%)	0 (0%)	0 (0%)
Race (n, % Black)	9 (47%)	4 (44%)	5 (56%)
Infant Sex (n, %Female)	10 (53%)	4 (44%)	3 (33%)
Preeclampsia with preterm delivery (n, %)			4 (44%)
Preeclampsia with SGA infants (n,%)			2 (22%)

Continuous variables are displayed as median (range); categorical variables are displayed as n (%). BMI, body mass index; BP, blood pressure. Early gestational BP: mean of all gestational blood pressures obtained prior to 20 weeks’. Preeclampsia with preterm delivery: preeclampsia with delivery before 37 weeks of gestation. Preeclampsia with SGA infants: preeclampsia with small-for-gestational-age (SGA) defined as birthweight percentile less than 10^th^.

^a^ Significant difference between preeclamptics and controls (P<0.05)

^b^ Significant difference between gestational hypertensives and controls (P<0.05)

^c^ Significant difference between preeclamptics and gestational hypertensives (P<0.05)

Group differences in continuous variables evaluated by Kruskal-Wallis with post hoc Dunn’s test; Categorical variables by Fisher’s Exact Test.

**Table 2:** Clinical characteristics of uncomplicated pregnancy (n = 25) and preeclampsia (n = 19) groups for placental evaluations.

	Uncomplicated Pregnancy (n = 25)	Preeclampsia (n = 19)	P-Value
Age	28 (20–38)	28 (17–36)	0.643
BMI pre-pregnancy (kg/m^2^) ^a^	27 (20–50)	27 (21–36)	0.611
Gestational weeks at delivery	39 (24–42)	33 (28–40)	<0.001
Early Gestational BP: ^b^			
Systolic	114 (58–127)	110 (98–127)	0.728
Diastolic	70 (61–110)	73 (57–77)	0.671
Pre-Delivery BP:			
Systolic	121 (50–137)	156 (130–177)	<0.001
Diastolic	72 (55–120)	95 (82–117)	<0.001
Baby Weight (grams)	3005 (525–3889)	1825 (561–3400)	0.001
Birth weight percentile	56 (11–94)	20 (0–93)	0.018
Placenta Weight (grams) ^c^	447 (214–380)	410 (130–660)	0.206
Uric Acid	N/M	6.59 (4.7–12.5)	-
Cigarette Smokers (n, %)	2 (8%)	1 (5%)	1.00
Race (n, % Black)	9 (36%)	6 (31%)	0.547
Infant Sex (n, % Female)	15 (60%)	10 (53%)	0.771
Antenatal steroids (n, %) ^d^	2 (9%)	12 (67%)	<0.001
Delivery by Cesarean section (n, %)	11 (44%)	7 (37%)	0.76
Cesarean with labor (n, %)	3 (27%)	5 (71%)	0.14
Labor (n, %) ^e^	13 (52%)	16 (84%)	0.05
Preeclampsia with preterm delivery (n, %)		17 (89%)	
Preeclampsia with SGA infants (n,%)		7 (37%)	

Continuous variables displayed as median (range); categorical variables displayed as n (%). P-values comparing preeclamptics vs. controls (Continuous: Mann-Whitney Test, Categorical: Fisher’s Exact Test); N/M: not measured.

^a^ Data missing for 1 women with preeclampsia.

^b^ Data missing for 1 woman with uncomplicated pregnancy and 5 women with preeclampsia.

^c^ Data missing for 11 women with uncomplicated pregnancy and 4 women with preeclampsia.

^d^ Data missing for 2 women with uncomplicated pregnancy and 1 woman with preeclampsia.

^e^ Number (percentage) of patients with labor at the time of delivery.

### Patient criteria

Women were prospectively recruited in the outpatient clinics or inpatient setting and all deliveries occurred at Magee-Womens Hospital. Patients with a history of chronic hypertension (determined by subject history and/or antihypertensive medication use prior to the pregnancy), diabetes, chronic renal disease or other significant metabolic disorders, current infection, fetal malformations or chromosomal abnormalities, multifetal gestation, or a positive toxicology screen were excluded. A panel of PEPP principal investigators and clinicians met monthly to adjudicate pregnancy outcomes. Controls were normotensive and without proteinuria throughout gestation, and delivered healthy babies in the absence of infection. Preeclampsia was defined as gestational hypertension with proteinuria, in accordance with standard criteria at the time (2000 National High Blood Pressure Education Program Working Group Report on High Blood Pressure in Pregnancy) [31]. Gestational hypertension was defined as persistent, new onset hypertension (systolic ≥ 140 mm Hg and/or diastolic ≥ 90 mm Hg) appearing after 20 weeks’ gestation and reverting post pregnancy. Threshold for proteinuria was ≥ 300 mg of protein in a 24-hour urine collection, a dipstick of 2+ or greater, a catheterized sample of 1+ or greater, or a protein to creatinine ratio ≥ 0.3. Our research-specific criteria for preeclampsia also included gestational hyperuricemia (>1 standard deviation above normal values for gestational age) [32]. The inclusion of hyperuricemia is thought to reduce the number of women misclassified as preeclamptic [32]. Women in the gestational hypertension outcome group manifested hypertension after gestational week 20 but not proteinuria.

Gestational age at delivery was based on last menstrual period and ultrasound, the latter available in most cases. Preterm birth was defined as delivery before 37 completed weeks of pregnancy. Gestational age-specific birth weight percentiles, adjusted for sex and race, were based upon data from Magee-Womens Hospital. Maternal race was by self-report at enrollment. Because of the small percentage of women who identified their race as other than black or white (<3%), results are reported as black or other. Pre-pregnancy body mass index [weight (kg)/height (m)^2^] was based on measured height and maternal self-report of pre-pregnancy weight at the initial clinic visit. Cigarette smoking status (any smoking since suspected pregnancy) was by self-report; these data were obtained at the time of sample collection; we previously demonstrated no significant discordance between self-report of smoking status and maternal plasma cotinine concentrations [33].

### Soluble syndecan-1

Blood samples were collected into sterile, ethylenediaminetetraacetic acid (EDTA)-containing collection tubes. The tubes were centrifuged for 20 min at 2,000 x g at room temperature portioned into aliquots and the plasma stored at −80°C without thaw until used. Concentrations of soluble Sdc1 in maternal plasma were measured in duplicate by ELISA (Diaclone sCD138 solid phase sandwich ELISA, Cell Sciences Inc., Canton, MA.) according to manufacturer’s instructions. The minimum detectable concentration was 4 ng/mL. The intra and inter-assay coefficients of variation were 6 and 10%, respectively. We performed sample dilution tests on separate pools of preeclampsia and uncomplicated pregnancy plasma; there was good linear correlation between the degree of sample dilution and measured Sdc1 concentrations, using 1:4, 1:8, and 1:16 (vol/vol) dilutions of each pool within the dynamic range of the assay (r^2^ = 0.90).

### Placental Immunohistochemical Staining and Scoring

We obtained placental biopsies immediately after elective Cesarean section or vaginal delivery from regions free of infarcts, between the margin and cord insertion. The villous tissue was cut into 0.5 cm^3^ pieces and rinsed with ice-cold phosphate buffered saline. After rinsing, portions of villous tissue were flash frozen and then placed into embedding medium (optimal cutting temperature compound, OCT, Tissue-Tek, Inc). Frozen OCT-embedded placentas were sectioned (7-μm), fixed in acetone, and peroxidase quenched by 3% H_2_O_2_. Acetone-fixed sections are appropriate for surface (CD) proteins [34]. Immunostaining was accomplished using the VECTASTAIN Elite ABC Kit, with anti-Sdc1 ectodomain antibody (H-174 rabbit anti- human polyclonal; sc-5632, Santa Cruz Biotechnology, Dallas TX) at 1:100 dilution, with blocking serum and 3, 3-diaminobenzidine supplied by the kit. Tissues were counterstained with hematoxylin (Vector Laboratories, Burlingame, CA). Negative controls were prepared by replacing the primary antibody with blocking serum. Bright field images were collected using a Nikon90i microscope and CCD camera (QImaging, Surrey, BC, Canada). Visual scoring of the STB of placental villi was performed on 4x magnification images, on a 0–4 semi-quantitative scale with 4 being the darkest stain along the entire apical surface. Images were scored by 2 technicians pre-trained to the scale but unaware of the source of the tissue. The weighted Cohen’s kappa coefficient for the 0–4 scoring between the technicians was 0.64, indicating “substantial” agreement. The values from each scorer were averaged.

### Western Blot Comparisons of Syndecan-1 in Placental Homogenates

Details are provided under Supporting Information (S1 Method). Twenty to thirty milligrams of frozen pulverized villous tissue was homogenized by sonication and further prepared for Western blot. Human recombinant CD138 (Diaclone ELISA, Cell Sciences Inc., Canton, MA) was used as a standard, and Precision Plus Protein Kaleidoscope Standards (Bio Rad Technologies, Hercules, CA) were used as molecular weight ladder. Blots were incubated with a rabbit polyclonal antibody against human Sdc1 ectodomain (H-174; Santa Cruz Biotechnology), followed by goat anti-rabbit IgG-HRP (Santa Cruz Biotechnology). Protein bands were detected by enhanced chemiluminescence. We used Amido Black as a loading control, as this total protein stain optimally reflects total protein concentration for semi-quantitative Western blot of placental homogenates [35]. The developed films were scanned into TIFF files and densitometry was carried out using digitizing software, UN-SCANIT™ Gel Version 4.3 (Silk Scientific Inc., Orem, UT).

### Isolation and Analysis of Placental RNA

Total cellular RNA was isolated from placental tissue with TRIzol reagent (Invitrogen, Carlsbad, CA) according to the manufacturer instructions. After isolation, double- and single-stranded DNA was removed by treatment with DNase I (Invitrogen). Purity of RNA was determined by measuring absorption at 280 and 260 nm. The cDNA was synthesized from 2 μg of RNA, converted to cDNA using an Applied Biosystems (Foster City, CA) high capacity cDNA kit. Control reactions without reverse transcriptase were performed to confirm the absence of DNA contamination. Quantitative real-time polymerase chain reaction (PCR) was performed on a TaqMan ABI PRISM 7700 sequence detector using 96-well optical plates (Applied Biosystems). Real-time PCR was performed using inventoried TaqMan Gene Expression Assays for syndecan-1 (Hs00896423_m1), syndecan-2 (Hs00299807_m1), glypican-1 (Hs00157805_m1) and 18s (Hs99999901_s1) (Applied Biosystems). The 18s gene expression assay was used as an endogenous reference for all samples. All amplification cycles were performed in a 50μl reaction in duplicate with cDNA equivalent to 100ng of total RNA. Relative quantification of the target expression was performed using the comparative cycle threshold (C_T_) method as described in Applied Biosystems User Bulletin #2, with normalization of the number of target gene copies (e.g., Sdc1) to an endogenous reference (18s) and relative to a calibrator (an uncomplicated pregnancy placenta).

### Statistical Analyses

Continuous data are expressed as median (range). Categorical data are given as frequencies (n) and percentages (%). Longitudinal changes in plasma soluble Sdc1 across normal pregnancy were evaluated using Repeated Measures Analysis of Variance on Ranks with post hoc Student-Newman-Keuls test. Non-parametric tests (Mann-Whitney U or Kruskal-Wallis with post hoc Dunn’s test) or Students’ t-test were used as appropriate to compare continuous data between groups. Between-group placental expression data were compared using ANCOVA on ranks, both with and without adjustment for gestational age. Fisher’s Exact test was used for categorical variables. Spearman’s rho correlations were computed to investigate bivariate relationships.

## Results

### Maternal plasma soluble Sdc1 concentrations across uncomplicated pregnancy

**Fig 1:**
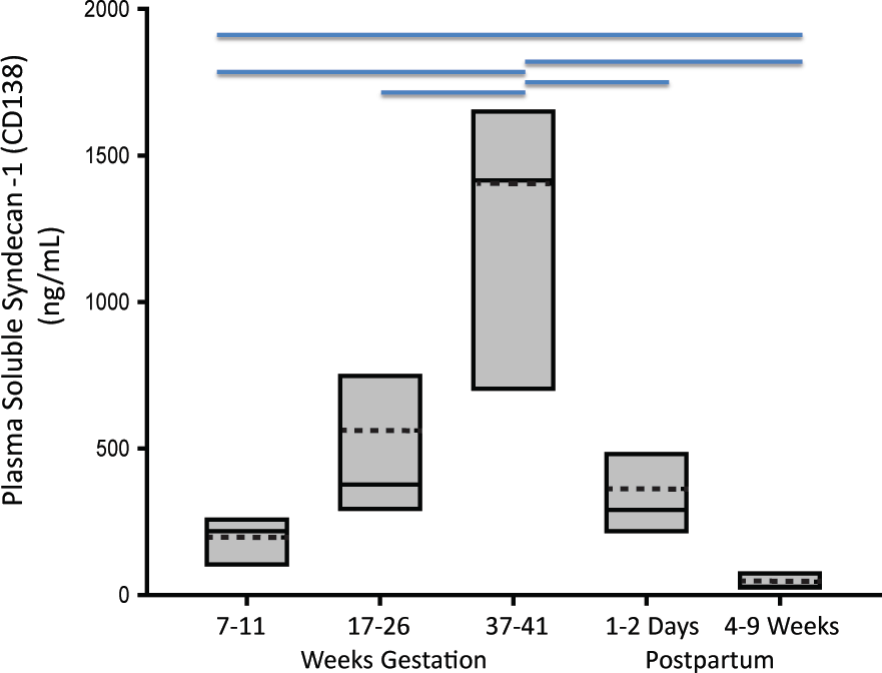
Soluble Sdc1 concentrations increased significantly with advancing gestation. Box-plot of soluble Sdc1 concentrations in maternal plasma as a function of successive gestational weeks (W) and postpartum stages, of n = 8 women with uncomplicated pregnancy outcome. The solid and dotted lines through the interior of the boxes correspond to median and mean values, respectively. The top and bottom of each box correspond to 75^th^ and 25^th^ percentiles, respectively. Horizontal lines on the top of the graph indicate significant differences between the time points (P<0.05; Repeated Measures Analysis of Variance on Ranks with post hoc Student-Newman-Keuls test).

Clinical characteristics are summarized in S1 Table. As shown in Fig 1, soluble Sdc1 concentrations increased significantly with advancing gestation, with highest values observed just prior to delivery [median ng/ml (range): 1415 (365–3000)], followed by a substantial decrease at 11–17 hours postpartum. With one exception (blood sample obtained 2 days before labor) the pre-delivery samples were obtained after labor onset. In additional samples between 27–29 weeks (before labor) from 4 patients, soluble Sdc1 was nearly double the corresponding value at 17–26 weeks (median mg/mL: 696 vs. 376), further indicative of progressive gestational increases. Soluble Sdc1 at 7–11 weeks of pregnancy [median ng/ml (range): 218 (102–290)] was approximately 7 times higher when compared to 4–9 weeks postpartum [28 (13–114) ng/mL; P<0.05] (Fig 1), the latter not different from values in the women with history of uncomplicated pregnancy assessed 1 year postpartum [27 (13–61) ng/mL; see postpartum data, below.].

### Mid-pregnancy comparison of soluble Sdc1

**Fig 2:**
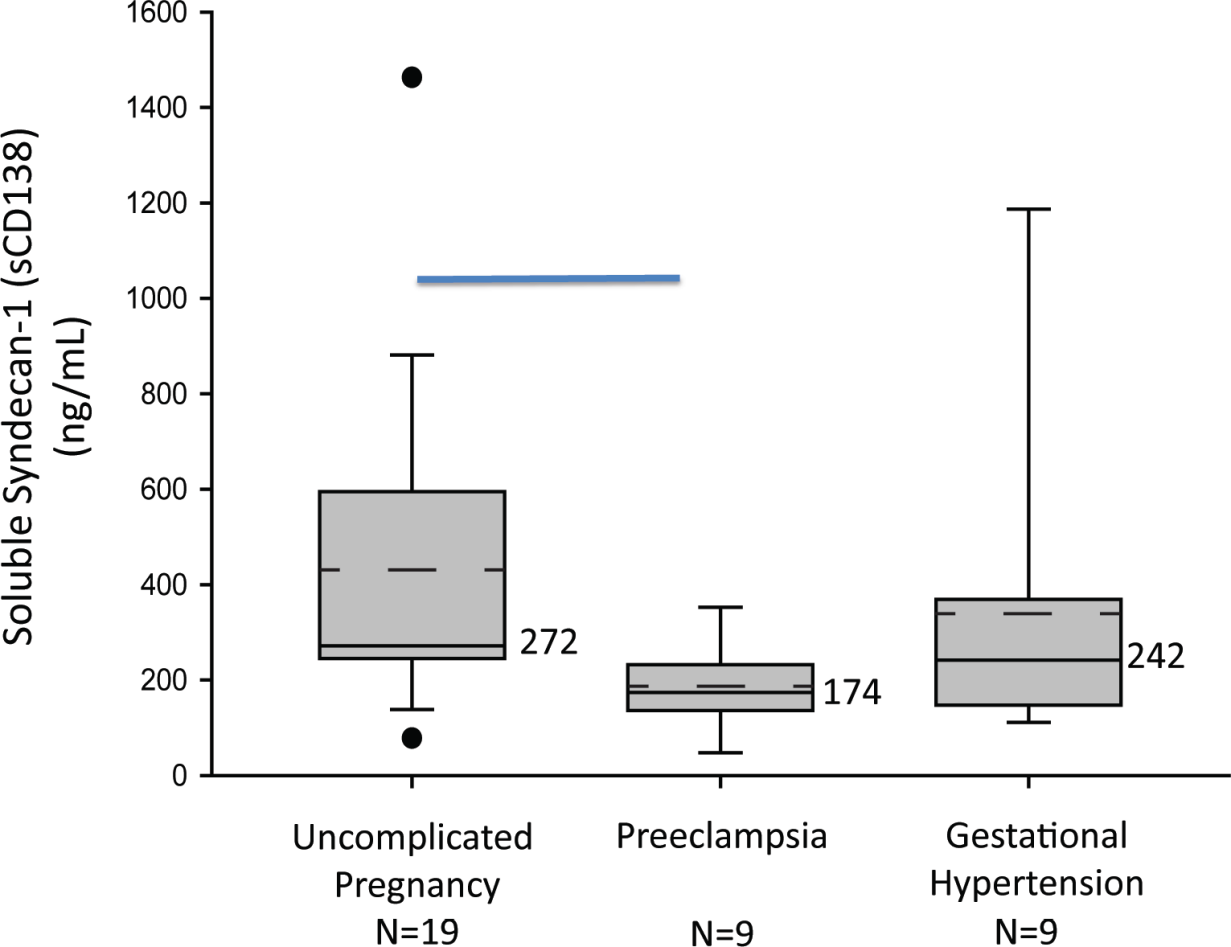
Plasma soluble Sdc1 is reduced at 20 weeks gestation in women who later develop preeclampsia. Box-plot of soluble Sdc1 concentrations in mid-pregnancy maternal plasma by pregnancy outcome group. The solid line through the interior of each box and the accompanying numerical value corresponds to the group median. The dotted line through the interior of each box denotes arithmetic mean. The top and bottom of each box correspond to 75^th^ and 25^th^ percentiles, respectively. The whiskers (t bars) on the bottom denote the 10^th^ percentile, and those on the top the 90^th^ percentile. Solid circles represent outlier values. The horizontal line, on the top of the graph indicate significant difference between control and preeclampsia groups (P<0.05; Kruskal-Wallis with Dunn’s Post-hoc test).

We compared soluble Sdc1 in gestational age-matched, mid-pregnancy (range 18–24 weeks) samples from 9 women who later developed preeclampsia, 9 who developed gestational hypertension (without proteinuria), and 19 with uncomplicated pregnancy. Clinical data are given in Table 1; all were non-smokers. There were no between-group differences in gestational age at the time of blood sampling, maternal age, body mass index, early gestational blood pressures, or racial distribution. All of the gestational hypertensive and uncomplicated pregnancy patients in this cohort delivered at term, with appropriate-for-gestational- age (AGA) infants. As shown in Fig 2, soluble Sdc1 concentrations were significantly lower, weeks before clinical development of preeclampsia [median ng/mL 174 (range 48–353)], but not gestational hypertension [242 (111–1187)], compared to controls [272 (78–1463)] (P<0.05, Kruskal-Wallis, post hoc Dunn’s). Examining the groups separately, we found no significant correlations of soluble Sdc1 with the clinical variables listed in Table 1.

Mid-gestation plasma soluble Sdc1 levels (at median gestational weeks 19.2 vs. 20.1, P = 0.74) were lower in women who later developed early-onset preeclampsia (gestational age at delivery <37 weeks; n = 4) compared to late-onset preeclampsia (gestational age at delivery ≥ 37 weeks; n = 5), but not significantly so [median ng/mL 163 (range 104–207) vs. 195 (158–251); P = 0.38]. The two preeclampsia patients who later delivered SGA infants had mid-gestation soluble Sdc1 values (161 and 217 ng/mL), straddling the overall preeclampsia group median (174 ng/mL).

### Cross-sectional 3^rd^ trimester study of soluble Sdc1

We compared 17 women with preeclampsia, with plasma obtained after disease onset, to 17 normotensive controls (S2 Table). The samples were obtained before any labor, and group gestational ages at the time of blood sampling were equivalent. The age, pre-pregnancy body mass index, early gestation blood pressures, percentage of cigarette smokers, and racial distribution did not differ between groups. Circulating soluble Sdc1 was ~2.5-fold lower in women with preeclampsia [median ng/mL (range): preeclampsia 281(101–2237) compared to controls 705 (243–2861); P<0.01]. The median birth weight percentile in the preeclampsia group was 11.3 (range 1–78). Eight of the 17 cases delivered babies that were small-for-gestational-age (SGA), defined as birth weight percentile <10. Median centile of this sub-group was 3 (range 1–9). Despite one high value outlier (2237 ng/mL), soluble Sdc1 levels in this SGA-preeclampsia sub-group were significantly lower than controls [median pg/mL (range): 225 (101–2237); P < 0.01]; these SGA-preeclampsia values, at gestational age 31 (range 26–33) weeks, were comparable to those observed at 7–11 weeks of uncomplicated pregnancy (Fig 1). In contrast, median soluble Sdc1 values in the preeclampsia sub-group with AGA infants [584 (159–1529)] only trended lower than controls (P = 0.18). The gestational age at time of blood sampling did not differ between controls and SGA-preeclampsia or AGA-preeclampsia.

In contrast, among plasma samples matched for gestational age at term prior to delivery (S3 Table), soluble Sdc1 in women with gestational hypertension [median ng/mL (range): 923 (443–1358)] did not differ significantly from controls [1308 (475–1637); P = 0.12].

Soluble Sdc1 correlated with infant birth weight percentile in women with gestational hypertension (r = 0.69, P<0.05) and their controls (r = 0.79, p<0.03), but the correlation was marginal in preeclamptic women (r = 0.45, P = 0.07) and not significant in their controls (r = 0.05, P = 0.84). No other correlations between soluble Sdc1 and clinical variables were found.

### Placental Sdc1 expression and plasma soluble Sdc1 in preeclampsia and controls

**Fig 3:**
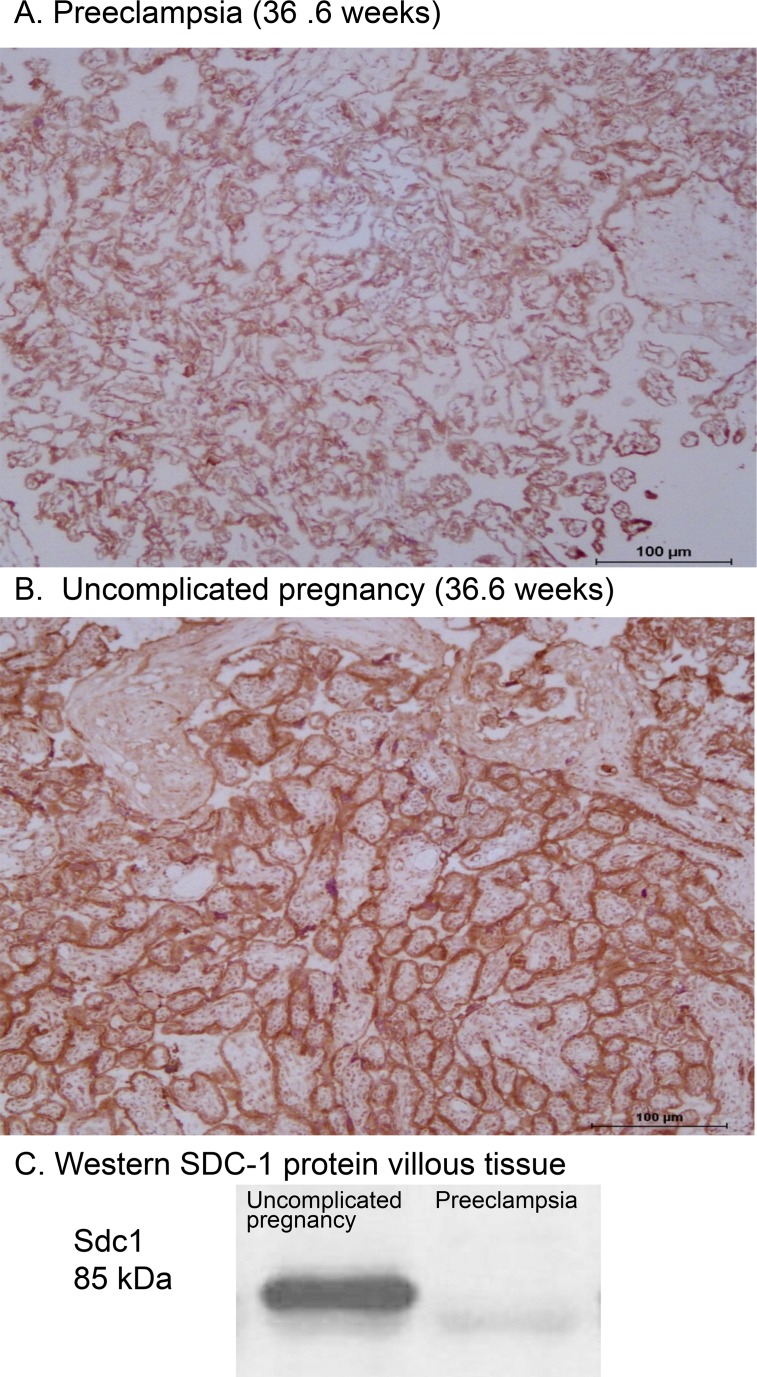
**Representative images of Sdc1 immunoreactivity in preeclampsia (****A****) compared to uncomplicated pregnancy (****B****) villous tissue.** Note the more intense staining on syncytiotrophoblast of uncomplicated pregnancy (immunohistochemical score = 4) compared to preeclampsia (score = 1), and apparent absence of staining in fetal villous vasculature throughout. Gestational age at delivery was 36.6 weeks for both placentas. C: Sample Western blot of villous tissue homogenates from the preeclampsia and control patients; band densities at the expected 85 kDa are consistent with reduced Sdc1 protein mass in preeclampsia. See also Table 3 for summary group data.

Preeclampsia and control placentas were evaluated; patient clinical characteristics are summarized in Table 2. Sdc1 immunoreactivity was prominent on villous syncytiotrophoblast but not detected on fetal villous vasculature (Fig 3). There was no discernable between-group difference in localization of the protein. The Sdc1 intensity score for Sdc1 on syncytiotrophoblast was significantly lower in preeclampsia (n = 19) [2.0 (1.0–3.5)] vs. controls (n = 25) [(3.0 (1.0–4.0)] (P<0.001, Unadjusted Rank ANCOVA). This difference remained significant (P<0.02) after adjusting for gestational age (Table 3). Presence or absence of labor did not appear to influence the Sdc1 immunostaining intensity score in either outcome group (data not shown), although the distribution of preeclampsia placenta samples collected in the presence (n = 16) vs. absence (n = 3) of labor was inadequate for meaningful assessment. Comparing the preeclampsia and uncomplicated pregnancy placentas exposed to labor, the intensity score for Sdc1 on syncytiotrophoblast was significantly lower in preeclampsia with labor (n = 16) [2.25 (2.0–3.0)] vs. controls with labor (n = 13) [(3.0 (2.5–4.0)] (P<0.03).

**Table 3:** Placental and pre-delivery plasma data, corresponding to the uncomplicated pregnancy (n = 25) and preeclampsia (n = 19) groups described in Supplementary S4 Table.

Outcomes	Uncomplicated Pregnancy (n = 25)	Preeclampsia (n = 19)	Unadjusted Rank ANCOVA	*Adjusted Rank ANCOVA
Sdc1 immunoreactivity score ^a^	3 (1–4)	2 (1–3.5)	<0.001	<0.02
Sdc1 expression, normalized to total protein (Amido Black)^b^	2.34 (1.41–4.37)	2.15 (1.23–2.73)	<0.05	0.126
Sdc1 mRNA ^c^^,^ ^d^	0.45 (0.08–8.47)	0.23 (0.11–1.57)	<0.01	<0.01
Syndecan-2 mRNA ^c^^,^ ^d^	0.6 (0.13–5.70)	0.21 (0.11–1.03)	0.06	0.09
Glypican-1 mRNA ^c^^,^ ^e^	0.94 (0.25–16.14)	0.52 (0.19–2.00)	0.295	0.552
Soluble Sdc1 (ng/mL) ^f^	1035 (171–3750)	495 (166–2236)	<0.02	0.254

Variables are displayed as median (range)

*Adjusted for gestational age

^a^ Immunoreactivity score: semi-quantitative ordinal (0 to 4+) scale by blinded observer, with absent staining considered scored as zero.

^b^ Data missing for 9 women with uncomplicated pregnancy and 3 women with preeclampsia.

^c^ Placental villous biopsy tissue mRNA content estimated by RT-PCR, comparative C_T_ method, with units relative to a normal pregnancy placenta = 1.0.

^d^ Data missing for 5 women with uncomplicated pregnancy and 3 women with preeclampsia.

^e^ Data missing for 6 women with uncomplicated pregnancy and 3 women with preeclampsia.

^f^ Data missing for 6 women with uncomplicated pregnancy and 2 women with preeclampsia.

We compared Sdc1 protein expression in villous tissue homogenates by Western blot, using all available samples from the same patients (n = 16 preeclampsia, 16 controls). By densitometry, median Sdc1 band intensities were lower in the preeclampsia group (P< 0.05; Table 3, Fig 3), consistent with the immunohistochemistry scores. However, differences were not significant after adjustment for gestational age (P = 0.13). Sdc1 mRNA expression level in the villous tissue homogenates was roughly half in preeclamptic women (n = 16) compared to controls (n = 20), significant both with and without adjustment for gestational age (Table 3). Syndecan-2 and glypican-1 mRNA did not differ by group.

Soluble Sdc1 was measured in pre-delivery maternal plasma, available from most of these patients (n = 17 preeclampsia, n = 19 controls). Soluble Sdc1 was lower in preeclampsia (P<0.02, Rank ANCOVA), but not after adjustment for gestational age (P = 0.20) (Table 3).

Sdc1 immunostaining score correlated with villous Sdc1 protein (by Western blot) in preeclamptic women (r = 0.52, P<0.05). Plasma soluble Sdc1 levels correlated with both villous Sdc1 protein (r = 0.54, P<0.03) and mRNA (r = 0.59, P<0.02) in controls. Pre-delivery systolic blood pressure correlated inversely (negatively) with immunostaining score in preeclamptic women (r = -0.50, P<0.05), with villous Sdc1 protein in controls (r = -0.75, P<0.001) and with soluble Sdc1 levels in controls (r = -0.54, p<0.03). Although not significant within each group separately, immunostaining score correlated significantly with birth weight percentile (r = 0.43, P<0.01) among all patients combined. Pre-pregnancy BMI correlated inversely with villous Sdc1 protein in both preeclamptic women (r = -0.54, P<0.04) and controls (r = -0.67, P<0.01). Gestational age at delivery correlated significantly with circulating soluble Sdc1 (preeclampsia r = 0.49, P<0.05; control: r = 0.64, P<0.01), and with villous mRNA in controls (r = 0.51, P<0.03).

### Soluble Sdc1 concentrations postpartum

We measured plasma soluble Sdc1 one year after completed pregnancy, comparing 17 non-pregnant women with a history of preeclampsia compared to 19 women with a history of uncomplicated pregnancy. These women were studied after first pregnancy, with the exception of two women with history of preeclampsia who were enrolled after their second completed pregnancy (one with recurrent preeclampsia, and one with preeclampsia in second pregnancy but unknown first pregnancy history). Study participants presented to the Magee-Womens Hospital Clinical and Translational Research Center in the morning after an overnight fast. At that time they completed a health history questionnaire, underwent a physical examination (weight, height, blood pressure, heart rate) and a urine test to rule out pregnancy, and a fasting blood sample was obtained. All women were non-lactating at the time, had no pre-pregnancy or current history of renal or vascular disease, and were nonsmokers (S4 Table). Plasma soluble Sdc1 concentrations were not different post-pregnancy [median ng/mL (range): women with a history of preeclampsia 23 (5–72) vs. control 27 (13–61); P = 0.98]. Soluble Sdc1 did not significantly differ post-pregnancy between women with a history of preterm vs. term preeclampsia, or between women with a history of preeclampsia with SGA vs. AGA infants (data not shown). We found no significant correlations of soluble Sdc1 with the clinical variables listed in S4 Table.

## Discussion

The glycocalyx is a negatively charged, gel-like mesh on the apical surface of epithelial and endothelial cells. The syncytiotrophoblast microvillous membrane surface has a substantial glycocalyx, but neither its normal functions nor its status/role in pregnancy disorders have been sufficiently evaluated. Both acute insults (e.g., ischemia/reperfusion, volume loading, heart surgery) and chronic conditions (e.g., sepsis, atherosclerosis and diabetes mellitus) can lead to damage to the vascular endothelial glycocalyx[36,37]. Shedding of the Sdc1 extracellular domain, and of other glycocalyx constituents such as hyaluronic acid and heparan sulfate, is normally regulated by matrix metalloproteinases and heparanases. However, shedding can be accentuated by tumor necrosis factor-alpha, oxidized lipoproteins, reactive oxygen species, and other inflammatory mediators [3,10,28,29].

Given evidence for a role of inflammation in the pathogenesis of preeclampsia we originally hypothesized that soluble Sdc1 would be elevated in women with the syndrome. Contrary to hypothesis, we found that soluble Sdc1 concentrations are significantly lower during the second trimester, ~16 weeks before clinically apparent preeclampsia, and in the 3^rd^ trimester after disease onset but before any labor, compared to controls with uncomplicated pregnancy. This pattern in gestational age-matched samples was not observed in women with gestational hypertension compared to controls, suggesting that reduced soluble Sdc1 is not simply a biomarker of hypertensive pregnancy. Although we cannot rule out the presence of subclinical disease at the time of blood sampling, these data suggest that Sdc1 dysregulation is an early event in preeclampsia pathogenesis.

Our study further shows that maternal plasma concentrations of soluble Sdc1 rise progressively with advancing pregnancy. By 7–11 weeks of pregnancy, concentrations were approximately 7-fold higher than values at 4–9 weeks after delivery. Soluble Sdc1 levels at term (median 1415 ng/mL) are similar to those reported during sepsis [10,38], and represent an approximate 50-fold increase over the postpartum levels. Concentrations normalized substantially toward early pregnancy levels within 24–48 hours postpartum. Our data do not preclude the possibility of labor and delivery (uterine contractions) causing a substantial increase in soluble Sdc1; systematic evaluation of this would require serial samples/measurements during the delivery. However, median soluble Sdc1 concentrations in the normotensive controls with 3rd trimester samples obtained before any labor (S2 Table; median gestational week 32.0) were roughly 25-fold higher than values in different controls measured at 4–9 weeks after delivery (Fig 1) or 1 year after delivery.

A prior study reported that serum Sdc1 concentrations rise with normal pregnancy (159-fold increase at term compared to nonpregnancy), with increases occurring mostly between weeks 20 and 30 of pregnancy[39]. However, that study reported even higher serum levels in patients with hemolysis, elevated liver enzymes and low platelets (HELLP) syndrome compared to normotensive controls matched by gestational age. Only two preeclampsia patients in our entire study manifested elevated liver enzymes and thrombocytopenia (hemolysis data not available); soluble Sdc1 values in these patients were below the preeclampsia median. The reason for the discrepancy between the two studies is not clear. However, 40% of the HELLP patients in the cited study did not fulfill diagnostic criteria for preeclampsia, and very few evidently had growth restricted/SGA infants as birth weights were on average 102 grams over that predicted on the basis of customized charts [39]. It is possible that HELLP syndrome as a distinct, severe disease involves widespread glycocalyx shedding with release of soluble glycans.

Using an antibody that recognizes the Sdc1 ectodomain (H-174), we found substantial expression of syndecan-1 on the STB of placental villi, but no detectable expression on fetal villous vasculature. This agrees with previous reports using several different monoclonal or polyclonal anti- Sdc1 antibodies, in which the apical surface of STB was extensively immunoreactive for Sdc1, but not evident on cytotrophoblast, stroma, extravillous trophoblast, or fetal vascular endothelium [21–27]. The rise in soluble Sdc1 with advancing pregnancy combined with the rapid decline in soluble Sdc1 levels within hours after delivery, and the robust expression of Sdc1 on STB in direct contact with maternal blood, suggest that the placenta is a major source of the high levels of soluble Sdc1 in the maternal circulation during pregnancy. Consistent with this hypothesis, we have observed that villous tissue fragments in explant culture produce appreciable amounts of soluble Sdc1 (~10 ng/mg tissue/24 hours) under 8%O_2_/5%C0_2_, 37°C incubation conditions (author’s unpublished data). However, this does not rule out maternal vasculature or blood cells as a significant source of soluble Sdc1 during pregnancy.

Our immunohistochemical, Western blot and mRNA data collectively indicate a decrease in placental Sdc1 expression with preeclampsia. The lack of matching for gestational age in the evaluation of preeclamptic and control placentas may be viewed as a limitation of our study. Seventeen of 19 preeclamptic placentas but only 2 of 25 controls in our placental analyses delivered preterm (gestational age <37 weeks). Nevertheless, group differences in immunostaining score and mRNA expression were significant after adjustment for gestational age. This agrees with previous reports of reduced expression of Sdc1 on STB microvillous membrane [21,22,25] or decreased Sdc1 in villous homogenates measured by ELISA [25], in which differences from controls were independent of gestational age. There is one contrasting report of an overall increase in expression of Sdc1 on STB, accompanied by significantly lower soluble Sdc1 concentrations in maternal serum, in preeclampsia (with or without HELLP syndrome) compared to normal controls[40]. Using an antibody (MI15) recognizing an extracellular epitope of the core protein, the authors reported focally reduced or absent Sdc1 immunoreactivity on the apical microvillous surface despite an overall increase in cytoplasmic Sdc1 in preeclampsia. When complicated with HELLP syndrome, however, the STB apical surface showed strong Sdc1 positivity. The authors suggested that abnormal cytoplasmic accumulation/retention of Sdc1 below the apical membrane occurs with preeclampsia, and that this might contribute to the observed reduction of soluble Sdc1 in maternal plasma [40]. We similarly used an anti-Sdc1 antibody directed against the extracellular domain but did not have the resolution to distinguish surface from cytoplasmic immunoreactivity. Our Western blot data using villous homogenates show less pronounced group differences in Sdc1 compared to our immunostaining (Table 3), possibly consistent with cytoplasmic versus membrane differences.

Our mid-pregnancy data are consistent with a possible contribution of soluble Sdc1 to the pathogenesis of preeclampsia. It has been hypothesized that both the reduction in circulating placental growth factor (PlGF) and the elevation of the antiangiogenic protein soluble fms-like tryosine kinase 1 [sFLT1; the soluble decoy receptor for PlGF and vascular endothelial growth factor (VEGF)]—that arise before clinically evident preeclampsia—are manifestations of syncytiotrophoblast cellular stress or syncytiotrophoblast dysfunction [41]. Although speculative at this point, the lower soluble Sdc1 in women destined to develop preeclampsia, and evidence of downregulation of syncytiotrophoblast Sdc1 in women with the syndrome, may be another expression of this stress response. The soluble Sdc1 ectodomain is known to function as a paracrine and autocrine effector or competitor, and may have relevant functions by influencing angiogenesis, coagulation pathways, inflammation, or lipid metabolism [14,20,28]. The shed Sdc1 ectodomain can compete with intact syndecan by sequestering heparan sulfate-binding ligands in the pericellular environment [28]. Syndecan-1 shedding is an important host response that facilitates the resolution of neutrophilic inflammation, potentially by aiding the clearance of pro-inflammatory chemokines. Soluble syndecans can act as extracellular chaperones; for example soluble VEGF-Sdc1 complexes can activate VEGF receptors on endothelial cells [14]. An excess of sFLT1 is thought to contribute to the pathogenesis of preeclampsia [42]. Membrane-bound heparan sulfate proteoglycans bind sFLT1 electrostatically, thereby regulating sFLT1 bioavailability [43]. Transgenic heparanase overexpression in mice significantly increases circulating sFLT1 [43]. Concentrations of plasma sFLT1 in non-pregnant women increase to late pregnancy levels shortly after intravenous heparin injection, suggesting that significant quantities of sFLT1 are normally bound to glycocalyx heparan sulfate proteoglycans [44]. However, the influence of membrane-bound or soluble Sdc1 on sFLT1 kinetics and activity has yet to be explored.

## Conclusions

Maternal plasma concentrations of shed (soluble) Sdc1 rise ~50-fold with gestation and revert postpartum. On average, women who later develop preeclampsia have lower levels of soluble Sdc1 in maternal plasma at 20 weeks’ gestation (before clinical disease onset) compared to women with uncomplicated pregnancy or gestational hypertension. Differences between women with preeclampsia compared to controls remain evident after disease onset, especially in preeclampsia with SGA neonates. Mirroring the differences in maternal circulation, preeclampsia is characterized by reduced expression Sdc1 on the syncytiotrophoblast of placental villi. There was no difference in concentration of soluble Sdc1 post pregnancy between women with prior preeclampsia and prior uncomplicated pregnancies. These data are collectively consistent with the placenta as a major source of soluble Sdc1, likely shed into the circulation at reduced quantity in women who develop preeclampsia. The study datasets were small and should be validated with larger cohorts. Because of the observational nature of our study we cannot establish a causal relationship between maternal plasma Sdc1 levels and the subsequent development of preeclampsia. Further studies are needed to determine the effects of altered Sdc1 kinetics on pregnancy physiology and its significance for placental and vascular glycocalyx integrity and function in normal pregnancies and those complicated by preeclampsia.

## Supporting Information

S1 Data Longitudinal Syndecan. (XLSX)

S2 Data 20 week Syndecan. (XLSX)

S3 Data Third Trimester Syndecan. (XLSX)

S4 Data Placenta and Syndecan. (XLSX)

S1 Method Western blots of Placental Homogenate. (DOCX)

S1 Table Clinical characteristics of uncomplicated pregnancy patients (n = 8), for measurement of soluble Sdc1 concentration in maternal plasma samples collected longitudinally during and after pregnancy. Continuous variables are given as median (range); categorical variables are displayed as n (%).
(DOCX)

S2 Table Clinical characteristics of uncomplicated pregnancy and preeclampsia groups for evaluation of soluble Sdc1 concentration in gestational age-matched 3^rd^ trimester maternal plasma samples. Continuous variables are displayed as median (range); categorical variables displayed as n (%). N/M: not measured. ^a^ Data missing for 1 woman with uncomplicated pregnancy and 5 women with preeclampsia.
(DOCX)

S3 Table Clinical characteristics of uncomplicated pregnancy and gestational hypertension groups for evaluation of soluble Sdc1 concentration in gestational age-matched 3^rd^ trimester maternal plasma samples. Continuous variables are displayed as median (range); categorical variables displayed as n (%). ^a^ Data missing for 1 woman with uncomplicated pregnancy.
(DOCX)

S4 Table Clinical characteristics; comparison study of plasma soluble Sdc1 concentrations 1 year after pregnancy. Continuous variables are given as median (range); categorical variables displayed as n(%).
(DOCX)
