# Supplementary material for: Low Soluble Syndecan-1 Precedes Preeclampsia
Source: PLoS One. 2016 Jun 14;11(6):e0157608. doi: 10.1371/journal.pone.0157608 (PMC4907460; doi:10.1371/journal.pone.0157608)
Supplement: S1 Method — (DOCX) [file pone.0157608.s005.docx]

**S1 Method. Western Blot Comparisons of Syndecan-1 in Placental Homogenates**

Twenty to thirty milligrams of frozen pulverized villous tissue from each patient was homogenized by sonication (Ultrasonic Processor, Tekmar, Cincinnati, OH) in RIPA buffer (50 mM Tris HCl, pH 8, 150 mM sodium chloride, 0.1% SDS, 0.5% sodium deoxycholate and 1% Triton X-100) containing 0.5 mM phenyl methyl sulfonyl fluoride (PMSF), 1mM sodium vanadate and 1x Halt Protease Inhibitor Cocktail (Thermo Scientific, Rockland,IL). The crude homogenate was centrifuged at 10,000x g at 4oC for 10 min. Protein estimation was carried out on the supernatant using the Pierce BCA protein assay (Rockford, IL). Samples were prepared in a fixed volume containing 10 µg protein, 5% ß-mercaptoethanol and Tris-glycine SDS sample buffer, boiled for 12 minutes then briefly centrifuged before loading onto gels. Human recombinant CD138 (Diaclone ELISA, Cell Sciences Inc., Canton, MA) was used as a standard, and Precision Plus Protein Kaleidoscope Standards (Bio Rad Technologies, Hercules, CA) were used as molecular weight ladder. The proteins were separated on 7.5% SDS-polyacrylamide gel and transferred to polyvinylidene fluoride membranes (Immobilon; Millipore, Bedford, MA) using a wet transfer system (Mini Trans-Blot Electrophoretic Transfer Cell, Bio-Rad). Detection of protein was carried out after blocking the membranes in a solution of 5% nonfat dry milk [in 10 mM Tris (pH 7.4), 150 mM NaCl (TBS)-0.05% Tween-20 (TBS-T)]. Blots were then incubated with a rabbit polyclonal antibody against human Sdc1 ectodomain (H-174; Santa Cruz Biotechnology) at a dilution of 1:500 in blocking solution overnight at 4oC. The blots were rinsed 3 times for 5 minutes each with TBS-T and incubated with goat anti-rabbit IgG-HRP (Santa Cruz Biotechnology) at 1:5000 in blocking solution for 1 h at room temperature. After appropriate washing, protein bands were detected by enhanced chemiluminescence (Santa Cruz Biotechnology) and exposed to x-ray film (Eastman Kodak Co, Rochester, NY). We used Amido Black as a loading control, given published data that this total protein stain optimally reflects total protein concentration for semi-quantitative Western blot of placental homogenates (35). To stain for Amido Black, the membranes were rewet in methanol, stained with a solution of 0.1% (w/v) amido black in 25% (v/v) isopropanol, and 10% (v/v) acetic acid for 10 minutes. They were destained by rinsing in deionized water and placed in 25% (v/v) isopropanol plus 10% (v/v) acetic acid for 10 minutes before air drying overnight. The developed films were scanned using a Hewlett Packard laser scanner (Scanjet 5370C, Hewlett Packard, Palo Alta, CA) into TIFF files in grey scale. Densitometry was carried out using automated digitizing software, UN-SCANIT™ Gel Version 4.3 (Silk Scientific Inc., Orem, UT).
