# Supplementary material for: Low Soluble Syndecan-1 Precedes Preeclampsia
Source: PLoS One. 2016 Jun 14;11(6):e0157608. doi: 10.1371/journal.pone.0157608 (PMC4907460; doi:10.1371/journal.pone.0157608)
Supplement: S1 Table — Continuous variables are given as median (range); categorical variables are displayed as n (%). (DOCX) [file pone.0157608.s006.docx]

**S1 Table. Clinical characteristics of uncomplicated pregnancy patients (n=8), for measurement of soluble Sdc1 concentration in maternal plasma samples collected longitudinally during and after pregnancy**

| Age (years)  BMI pre-pregnancy (kg/m^2^) | 24 (21 - 28)  29 (24 – 35) |
| --- | --- |
| Gestational weeks at delivery | 40 (39 – 41) |
| Early gestational BP (<20wks.)  Systolic (mm Hg)  Diastolic (mm Hg)  Pre-delivery BP:  Systolic (mm Hg)  Diastolic (mm Hg) | 118 (103 – 124)  68 (63 – 76)  129 (119 – 139)  75 (69 – 79) |
| Baby weight (g) | 3770 (2773 – 4104) |
| Birth weight percentile | 69 (27 – 87) |
| Cigarette smokers (n, %) | 3 (37%) |
| Race (n, % Black) | 2 (25%) |
| Infant Sex (n, % Female) | 5 (63%) |

Continuous variables are given as median (range); categorical

variables are displayed as n (%).
