# Supplementary material for: Low Soluble Syndecan-1 Precedes Preeclampsia
Source: PLoS One. 2016 Jun 14;11(6):e0157608. doi: 10.1371/journal.pone.0157608 (PMC4907460; doi:10.1371/journal.pone.0157608)
Supplement: S2 Table — Continuous variables are displayed as median (range); categorical variables displayed as n (%). N/M: not measured. a Data missing for 1 woman with uncomplicated pregnancy and 5 women with preeclampsia. (DOCX) [file pone.0157608.s007.docx]

**S2 Table. Clinical characteristics of uncomplicated pregnancy and preeclampsia groups for evaluation of soluble Sdc1 concentration in gestational age-matched 3^rd^ trimester maternal plasma samples**

|  | Uncomplicated  Pregnancy (n=17) | Preeclampsia  (n=17) | P value |
| --- | --- | --- | --- |
| Age (years)  BMI pre-pregnancy (kg/m^2^) | 26 (17-35)  25 (17-40) | 28 (17-44)  27 (22-35) | 0.63  0.17 |
| Gestational weeks at venipuncture  Gestational weeks at delivery | 32.0 (27.7-40.6)  40 (37-42) | 32.6 (25.6-41.7)  33 (27-42) | 0.54  <0.001 |
| Early gestational BP (<20wks.) ^a^  Systolic (mm Hg)  Diastolic (mm Hg)  Pre-delivery BP:  Systolic (mm Hg)  Diastolic (mm Hg) | 109 (98-126)  68 (60-84)  119 (105-136)  73 (40-88) | 115 (98-123)  72 (57-77)  163 (130-177)  98 (83-117) | 0.11  <0.05  <0.001  <0.001 |
| Birth weight percentile | 49 (14-100) | 11 (1-78) | <0.01 |
| Uric acid (mg/dL) | N/M | 6.8 (4.8-8.8) | --- |
| Cigarette smokers (n, % smokers) | 4 (24%) | 3 (18%) | 0.69 |
| Race (n, % Black) | 4 (24%) | 5 (30%) | 0.56 |
| Infant Sex (n, % Female) | 8 (47%) | 11 (65%) | 0.49 |
| Antenatal steroids (n, %) | 0 (0%) | 14 (82%) | <0.001 |
| Labor at time of venipuncture (n, %) | 0 (0%) | 0 (0%) | 1.0 |

Continuous variables are displayed as median (range); categorical variables displayed as n (%). N/M: not measured.

^a^ Data missing for 1 woman with uncomplicated pregnancy and 5 women with preeclampsia.
