# Supplementary material for: Low Soluble Syndecan-1 Precedes Preeclampsia
Source: PLoS One. 2016 Jun 14;11(6):e0157608. doi: 10.1371/journal.pone.0157608 (PMC4907460; doi:10.1371/journal.pone.0157608)
Supplement: S3 Table — Continuous variables are displayed as median (range); categorical variables displayed as n (%). a Data missing for 1 woman with uncomplicated pregnancy. (DOCX) [file pone.0157608.s008.docx]

**S3 Table. Clinical characteristics of uncomplicated pregnancy and gestational hypertension groups for evaluation of soluble Sdc1 concentration in gestational age-matched 3^rd^ trimester maternal plasma samples**

|  | Uncomplicated  Pregnancy  (n=8) | Gestational Hypertension (n=8) | P value |
| --- | --- | --- | --- |
| Age (years)  BMI pre-pregnancy (kg/m^2^) | 26 (19-38)  25 (20-32) | 31 (19-39)  25 (17-46) | 0.55  0.80 |
| Gestational weeks at venipuncture  Gestational weeks at delivery | 39.6 (38.0-40.5)  39.7 (38.0-40.6) | 38.9 (36.0-41.0)  39.6 (38.6-41.4) | 0.51  0.57 |
| Early gestational BP (<20wks.) ^a^  Systolic (mm Hg)  Diastolic (mm Hg)  Pre-delivery BP:  Systolic (mm Hg)  Diastolic (mm Hg) | 115 (100-125)  70 (61-74)  127 (114-136)  72 (66-83) | 115 (107-128)  70 (57-75)  147 (120-162)  87 (76-107) | 0.36  0.93  <0.001  <0.001 |
| Birth weight percentile | 40 (17-99) | 48 (11-89) | 0.96 |
| Uric acid (mg/dL) | ---- | 5.1 (3.2-6.0) | --- |
| Cigarette smokers (n, % smokers) | 2 (25%) | 0 (0%) | 0.45 |
| Race (n, % Black) | 1 (13%) | 2 (25%) | 1.0 |
| Infant Sex (n, % Female) | 5 (63%) | 7 (88%) | 0.57 |
| Antenatal steroids (n, %) | 0 (0%) | 0 (0%) | 1.0 |
| Labor at time of venipuncture (n, %) | 6 (75%) | 2 (25%) | 0.13 |

Continuous variables are displayed as median (range); categorical variables displayed as n (%).

^a^ Data missing for 1 woman with uncomplicated pregnancy.
