# Supplementary material for: Low Soluble Syndecan-1 Precedes Preeclampsia
Source: PLoS One. 2016 Jun 14;11(6):e0157608. doi: 10.1371/journal.pone.0157608 (PMC4907460; doi:10.1371/journal.pone.0157608)
Supplement: S4 Table — Continuous variables are given as median (range); categorical variables displayed as n(%). (DOCX) [file pone.0157608.s009.docx]

**S4 Table. Clinical characteristics; comparison study of plasma soluble Sdc1 concentrations 1 year after pregnancy**

| Mean (±SD) or median (interquartile range) | Prior uncomplicated pregnancy  (n=19) | Prior preeclampsia  (n=17) | P- value |
| --- | --- | --- | --- |
| **Post-pregnancy Data** |  |  |  |
| Age (years) | 23 (19-38) | 29 (18-43) | 0.20 |
| Body mass index (kg/m2) | 27 (18-44) | 30 (17-43) | 0.51 |
| Days post delivery | 324 (208-708) | 379 (202-592) | 0.74 |
| Systolic BP (mm Hg) | 110 (100-124) | 120 (90-178) | 0.08 |
| Diastolic BP (mm Hg) | 70 (53-90) | 78 (56-107) | 0.15 |
| Oral Contraceptive Use (n, %) | 10 (53%) | 6 (35%) | 0.34 |
|  |  |  |  |
| **Pregnancy Data** |  |  |  |
| Gestational weeks at delivery  Pre-delivery BP:  Systolic (mm Hg)  Diastolic (mm Hg) | 39.6 (38.9-40.4)^a^  127 (119-135)^a^  75 (72-81)^a^ | 37.7 (35.7-39.0)^b^  150 (144-162)^b^  95 (87-99)^b^ | <0.05  <0.001  <0.001 |
|  |  |  |  |
| Birth weight percentile | 69 (27-89)^a^ | 29 (9-42)^b^ | <0.05 |
| Uric Acid (mg/dL) | N/M | 5.8 (5.6-6.5)^b^ |  |
| Preeclampsia with preterm delivery (n, %)  Preeclampsia with SGA infants (n, %) |  | 6 (37%)^b^  4 (25%)^b^ |  |
| **Baseline Data** |  |  |  |
| BMI pre-pregnancy (kg/m^2^)  Race (n, % Black) | 27 (22-33)^a^  6 (32%) | 27 (23-34)^c^  7 (41%) | 0.75  0.73 |
|  |  |  |  |

Continuous variables are given as median (range); categorical variables displayed as n (%).

^a^ Data missing for 2 women with prior uncomplicated pregnancy

^b^ Data missing for 1 woman with prior preeclampsia

^c^ Data missing for 2 women with prior preeclampsia
